# Supplementary material for: Clinical Decision Support Systems for Antibiotic Prescribing: An Inventory of Current French Language Tools
Source: Antibiotics (Basel). 2022 Mar 14;11(3):384. doi: 10.3390/antibiotics11030384 (PMC8944435; doi:10.3390/antibiotics11030384)
Supplement: Supplementary file 1 [file antibiotics-11-00384-s001.zip › antibiotics-1601900-supplementary.pdf]

antibiotics

MDPI

Supplementary Materials

Table S1. Search strategy for Pubmed.

Keywords and MeshWords for PubMed	
CDSS	("Decision Support Systems, Clinical"[Mesh] OR "CDSS"[tiab] OR "computerized decision support system"[tiab] OR "clinical decision support system"[tiab] OR "clinical decision support"[tiab] OR "computer-aided decision support"[tiab] OR "computer-assisted decision support"[tiab] OR "smartphone application"[tiab]
Antibiotics	"Anti-Bacterial Agents"[Mesh] OR "Anti-Infective Agents"[Mesh] OR Antibiotic*[tiab] OR Anti-biotic*[tiab] OR Antimicrobial*[tiab] OR Anti-microbial*[tiab] OR Antibacterial*[tiab] OR Anti-bacterial*[tiab] OR Antiinfective*[tiab] OR Anti-infective*[tiab]
French-language	"France" OR "French"

Table S2. Characteristics of *Antibioclic.*

Antibioclic			
Administration			
Name			Comité Antibioclic
	Funding	Nature	Public
		Name(s)	- French Infectious Diseases Society (SPILF)<br>- French College of General Medicine (CMG)<br>- French College of Teaching General Practitioners (CNGE)
Scientific references			Guidelines from scientific societies, French Infectious Diseases Society (SPILF), College of Professors of Infectious and Tropical Diseases (CMIT), French National Authority for Health (HAS)
Date of creation			2011
	Updating	Last	April 2021
		Frequency	Monthly
Contact			contact.antibioclic@gmail.com
Access			
	Cost		Free
	Smartphone app		Yes
		iOS	Yes
		Downloads (n)	N/A
		Reviews (n)	25
		Rating (/5)	5
		Android	Yes
		Downloads (n)	N/A
		Reviews (n)	138
		Rating (/5)	4,5
	Website		Yes
	Website URL	https://antibioclic.com/	
		Users/day (n)	5365 (2018)
		Requests/day (n)	11 125 (2018)
	Offline mode		Yes
	Software		No
	Local parameterization possible		No

Developer	No
User	No
Users	
Primary care	Yes
Dental medicine	No
Hospital medicine	No
Surgical prophylaxis	No
Patients	
Children	Yes
Pregnant women	Yes
Breastfeeding	Yes
Chronic kidney disease	Yes
Informations required	
Type of infection	Urinary tract infections<br>Genital infections<br>Upper respiratory tract infections<br>Lower respiratory tract infections<br>Skin and soft tissues infections<br>Digestive tract infections<br>Prophylaxis<br>COVID-19
Systematic criteria	Site of infection<br>Nature of infection<br>Patient profile (Adults/Children)
Optional criteria	Pregnant women<br>Breastfeeding<br>Chronic Kidney Disease<br>Additionnal information about infection
Type of information provided	
Antibiotic selection	Yes
Priority	Yes
Dose	Yes
Route of administration	Yes
Duration	Yes
Allergy	Yes
Side effects	No
Adaptation GFR	Yes
Prescription printing	Only on Antibioclic+ (additionnal service for users in the Parisian area)
Context and reminders	Yes
Locally adapted	No
Scientific sources cited	Yes
References	- Jeanmougin et al. [Antibioclic: a tool for rational antibiotic therapy in primary care]. *Rev Prat* 2012<br><br>- Delory et al. [A computerized decision support system (CDSS) for antibiotic prescription in primary care—Antibioclic: implementation, adoption and sustainable use in the era of extended antimicrobial resistance]. *Journal of Antimicrobial Chemotherapy* 2020
Description	CDSS description<br>Implementation process and utilization

Table S3. Characteristics of *Antibiogarde.*

**Antibiogarde**			
Administration			
Name			Association des Auteurs d'ANTIBI-OGARDE
	Funding	Nature	Public
		Name(s)	French National Authority for Health (HAS)
Scientific references			National guidelines mainly from French Infectious Diseases Society (SPILF), College of Professors of Infectious and Tropical Diseases (CMIT), French Society of Anesthesia and Intensive Care Medecine (SFAR), French Intensive Care Society (SRLF), French Society of Respiratory Diseases (SPLF) and French National Authority for Health (HAS)
Date of creation			2003
	Updating	Last	2020
		Frequency	Yearly
Contact			contact@antibiogarde.org
Access			
	Cost		7,99 € per year for website<br>9,99 € per year for iOS app
	Smartphone app		Yes
		iOS	Yes
		Downloads (n)	N/A
		Reviews (n)	2
		Rating (/5)	5
		Android	No
		Downloads (n)	N/A
		Reviews (n)	N/A
		Rating (/5)	N/A
	Website		Yes
	Website URL	https://webhopital.antibiogarde.org/login	
		Users/day (n)	N/A
		Requests/day (n)	N/A
	Offline mode		Yes
	Software		Yes (via hospital Intranet)
	Local parameterization possible		Yes
		Developer	Yes
		User	N/A
Users			
Primary care			Yes
Dental medicine			Yes
Hospital medicine			Yes
Surgical prophylaxis			No
Patients			
Children			Yes (incomplete)
Pregnant women			Yes (incomplete)

Breastfeeding	No
Chronic kidney disease	Yes
Informations	
Type of infection	Urinary tract infections<br>Genital infections<br>Upper respiratory tract infections<br>Lower respiratory tract infections<br>Skin and soft tissues infections<br>Digestive tract infections<br>Central nervous system infections<br>Bone and joint infections<br>Eye infections<br>Dental infections<br>Febrile neutropenia
Systematic criteria	Site of infection<br>Nature of infection
Optional criteria	Additionnal information about infection or patient
Type of information provided	
Antibiotic selection	Yes
Priority	Yes
Dose	Yes
Route of administration	Yes
Duration	Yes
Allergy	Yes
Side effects	No
Adaptation GFR	Yes
Prescription printing	No
Context and reminders	Yes
Locally adapted	No
Scientific sources cited	Yes
References	No
Description	N/A

Table S4. Characteristics of *Antibiogilar.*

**Antibiogilar**		
Administration		
Name		Groupement d'Infectiologie et de Lutte contre l'Antibio Résistance (GILAR)
Funding	Nature	Public
	Name(s)	- ARS Hauts-de-France<br>- Groupement d'Infectiologie et de Lutte contre l'Antibio Résistance
Scientific references		Local and national guidelines from French Infectious Diseases Society (SPILF), French National Agency for the Safety of Medicines (ANSM), French National Authority for Health (HAS)
Date of creation		February 2019
Updating	Last	May 2021
	Frequency	Monthly
Contact		antibiogilar@gmail.com
Access		
Cost		Free

Smartphone app		Yes
	iOS	Yes
	Downloads (n)	N/A
	Reviews (n)	5
	Rating (/5)	4,8
	Android	Yes
	Downloads (n)	N/A
	Reviews (n)	11
	Rating (/5)	4,5
Website		Yes
Website URL	https://www.gilar.org/antibiogilar	
	Users/day (n)	N/A
	Requests/day (n)	N/A
Offline mode		Yes
Software		No
Local parameterization possible		Yes
	Developer	Yes
	User	N/A
Users		
Primary care		Yes
Dental medicine		No
Hospital medicine		Yes
Surgical prophylaxis		Yes
Patients		
Children		Yes
Pregnant women		Yes (incomplete)
Breastfeeding		No
Chronic kidney disease		Yes
Informations		
Type of infection	Urinary tract infections<br>Genital infections<br>Upper respiratory tract infections<br>Lower respiratory tract infections<br>Skin and soft tissues infections<br>Digestive tract infections<br>Prophylaxis<br>Central nervous system infections<br>Cardiovascular infections<br>Bone and joint infections<br>Febrile neutropenia<br>COVID-19	
Systematic criteria	Site of infection<br>Nature of infection	
Optional criteria	Additionnal information about infection or patient	
Type of information provided		
Antibiotic selection	Yes	
Priority	Yes	
Dose	Yes	
Route of administration	Inconstant	
Duration	Yes	
Allergy	Yes	
Side effects	No	
Adaptation GFR	Yes	
Prescription printing	No	
Context and reminders	Yes	

Locally adapted	Yes
Scientific sources cited	Yes
References	No
Description	N/A

Table S5. Characteristics of *antibioGUIDE* (Basse-Normandie).

**antibioGUIDE (Basse-Normandie)**			
Administration			
Name			Omedit Basse-Normandie<br>ARS Basse-Normandie<br>CClin-ARlin Basse-Normandie
	Funding	Nature	Public
		Name(s)	ARS Basse-Normandie
Scientific references			International and national guidelines from French Infectious Diseases Society (SPILF), College of Professors of Infectious and Tropical Diseases (CMIT), French National Authority for Health (HAS), Infectious Diseases Society of America (IDSA), French National Agency for the Safety of Medicines (ANSM)
Date of creation			April 2015
	Updating	Last	July 2015
		Frequency	< yearly
Contact			ars-bnormandie-omedit@ars.sante.fr
Access			
	Cost		Free
	Smartphone app		Yes
		iOS	Yes
		Downloads (n)	N/A
		Reviews (n)	N/A
		Rating (/5)	N/A
		Android	Yes
		Downloads (n)	N/A
		Reviews (n)	N/A
		Rating (/5)	N/A
	Website		Yes
	Website URL	http://antibioguide.free.fr/	
		Users/day (n)	N/A
		Requests/day (n)	N/A
	Offline mode		Yes
	Software		No
	Local parameterization possible		No
		Developer	No
		User	No
Users			
Primary care			Yes
Dental medicine			No
Hospital medicine			Yes
Surgical prophylaxis			No
Patients			
Children			Yes (incomplete)
Pregnant women			Yes (incomplete)

Breastfeeding		No
Chronic kidney disease		No (refers to GPR website)
Informations		
Type of infection	Urinary tract infections<br>Genital infections<br>Upper respiratory tract infections<br>Lower respiratory tract infections<br>Skin and soft tissues infections<br>Digestive tract infections<br>Central nervous system infections<br>Bone and joint infections<br>Febrile neutropenia	
Systematic criteria	Site of infection<br>Nature of infection	
Optional criteria	Additionnal information about patient or infection	
Type of information provided		
Antibiotic selection	Yes	
Priority	Inconstant	
Dose	Yes	
Route of administration	Inconstant	
Duration	Yes	
Allergy	Yes	
Side effects	No	
Adaptation GFR	No	
Prescription printing	No	
Context and reminders	No	
Locally adapted	No	
Scientific sources cited	Yes	
References	No	
Description	N/A	

Table S6. Characteristics of *Antibioguide* (Perpignan).

**Antibioguide (Perpignan)**		
Administration		
Name		Commission des anti-infectieux du CH de Perpignan
Funding	Nature	Public
	Name(s)	N/A
Scientific references		National guidelines from French Infectious Diseases Society (SPILF), French National Agency for the Safety of Medicines (ANSM), French National Authority for Health (HAS)
Date of creation		2018
Updating	Last	January 2020
	Frequency	Yearly
Contact		aurelia.eden@ch-perpignan.fr
Access		
Cost		Free
Smartphone app		Yes
	iOS	No
	Downloads (n)	N/A
	Reviews (n)	N/A

	Rating (/5)	N/A
	Android	Yes
	Downloads (n)	N/A
	Reviews (n)	8
	Rating (/5)	5
Website		No
	Users/day (n)	N/A
	Requests/day (n)	N/A
Offline mode		No
Software		No
Local parameterization possible		No
	Developer	No
	User	No
Users		
Primary care		Yes
Dental medicine		No
Hospital medicine		Yes
Surgical prophylaxis		No
Patients		
Children		Yes (incomplete)
Pregnant women		Yes (incomplete)
Breastfeeding		No
Chronic kidney disease		No
Informations		
Type of infection	Urinary tract infections<br>Genital infections<br>Upper respiratory tract infections<br>Lower respiratory tract infections<br>Skin and soft tissues infections<br>Digestive tract infections<br>Central nervous system infections<br>Bone and joint infections<br>Febrile neutropenia	
Systematic criteria	Site of infection<br>Nature of infection	
Optional criteria	Additionnal information about infection or patient	
Type of information provided		
Antibiotic selection	Yes	
Priority	Yes	
Dose	Yes	
Route of administration	Yes	
Duration	Yes	
Allergy	Yes	
Side effects	No	
Adaptation GFR	No	
Prescription printing	No	
Context and reminders	No	
Locally adapted	No	
Scientific sources cited	No	
References	No	
Description	N/A	

Table S7. Characteristics of *AntibioEst.*

**AntibioEst**		
Administration		
Name		Association AntibioEst
Funding	Nature	Public
	Name(s)	- ARS Grand-Est<br>- Association AntibioEst
Scientific references		European and national guidelines from scientic societies, French Infectious Diseases Society (SPILF), French National Authority for Health (HAS)
Date of creation		2018
Updating	Last	June 2021
	Frequency	Yearly
Contact		antibioest@chru-nancy.fr
Access		
Cost		Free
Smartphone app		Yes
	iOS	Yes
	Downloads (n)	N/A
	Reviews (n)	5
	Rating (/5)	4,8
	Android	Yes
	Downloads (n)	N/A
	Reviews (n)	5
	Rating (/5)	4,2
Website		Yes
Website URL	https://guides.antibioest.org/	
	Users/day (n)	N/A
	Requests/day (n)	N/A
Offline mode		No
Software		No
Local parameterization possible		No
	Developer	No
	User	No
Users		
Primary care		Yes
Dental medicine		Yes
Hospital medicine		Yes
Surgical prophylaxis		Yes
Patients		
Children		Yes
Pregnant women		Yes (incomplete)
Breastfeeding		No
Chronic kidney disease		Yes
Informations		
Type of infection		Urinary tract infections<br>Genital infections<br>Upper respiratory tract infections<br>Lower respiratory tract infections<br>Skin and soft tissues infections<br>Digestive tract infections<br>Prophylaxis<br>Central nervous system infections

	Cardiovascular infections<br>Bone and joint infections<br>Febrile neutropenia<br>Bloodstream infections<br>Eye infections<br>Dental infections
Systematic criteria	Site of infection<br>Nature of infection
Optional criteria	Additionnal information about infection, pathogen or patient
Type of information provided	
Antibiotic selection	Yes
Priority	Yes
Dose	Yes
Route of administration	Yes
Duration	Yes
Allergy	Yes
Side effects	No
Adaptation GFR	Yes
Prescription printing	No
Context and reminders	Yes
Locally adapted	No
Scientific sources cited	Yes
References	No
Description	N/A

Table S8. Characteristics of *APPLIBIOTIC.*

**APPLIBIOTIC**			
Administration			
Name			Polyclinique Bordeaux Nord
Funding		Nature	Private
		Name(s)	Polyclinique Bordeaux Nord
Scientific references			Local and national guidelines from French scientific societies (SFAR, SRLF, SPILF, CNGOF, SFRP, GPPIP, GRIOGO), French National Agency for the Safety of Medicines (ANSM), French National Authority for Health (HAS)
Date of creation			March 2017
Updating		Last	October 2019
		Frequency	Variable
Contact			applibiotic@bordeauxnord.com
Access			
	Cost		Free
	Smartphone app		Yes
		iOS	Yes
		Downloads (n)	N/A
		Reviews (n)	4
		Rating (/5)	5
		Android	Yes
		Downloads (n)	N/A
		Reviews (n)	21
		Rating (/5)	4,8

	Website	No
	Users/day (n)	N/A
	Requests/day (n)	N/A
	Offline mode	Yes
	Software	No
	Local parameterization possible	No
	Developer	No
	User	No
Users		
	Primary care	Yes
	Dental medicine	Yes
	Hospital medicine	Yes
	Surgical prophylaxis	Yes
Patients		
	Children	Yes
	Pregnant women	Yes (incomplete)
	Breastfeeding	No
	Chronic kidney disease	No
Informations		
Type of infection	Urinary tract infections<br>Genital infections<br>Upper respiratory tract infections<br>Lower respiratory tract infections<br>Skin and soft tissues infections<br>Digestive tract infections<br>Prophylaxis<br>Central nervous system infections<br>Cardiovascular infections<br>Bone and joint infections<br>Febrile neutropenia<br>Eye infections<br>Central venous catheter-related infections<br>Dental infections	
Systematic criteria	Site of infection<br>Nature of infection	
Optional criteria	Additionnal information about infection, pathogen or patient	
Type of information provided		
Antibiotic selection	Yes	
Priority	Yes	
Dose	Yes	
Route of administration	Yes	
Duration	Yes	
Allergy	Yes	
Side effects	No	
Adaptation GFR	No	
Prescription printing	No	
Context and reminders	Yes	
Locally adapted	No	
Scientific sources cited	Yes	
References	No	
Description	N/A	

Table S9. Characteristics of *ePOPI.*

**ePOPI**		
Administration		
Name		College of Professors of Infectious and Tropical Diseases (CMIT)
Funding	Nature	Public
	Name(s)	College of Professors of Infectious and Tropical Diseases (CMIT)
Scientific references		International, european and national guidelines from World Heath Organization (WHO), European Medicines Agency (EMA), French National Agency for the Safety of Medicines (ANSM), French Public Health Council (HSCP), French National Authority for Health (HAS) and scientific societies
Date of creation		2014
Updating	Last	July 2021
	Frequency	Monthly
Contact		contact@epopi.fr
Access		
Cost		33,00 € per year
Smartphone app		Yes
	iOS	Yes
	Downloads (n)	N/A
	Reviews (n)	5
	Rating (/5)	2,4
	Android	Yes
	Downloads (n)	N/A
	Reviews (n)	11
	Rating (/5)	3,5
Website		Yes
Website URL	https://epopi.fr/	
	Users/day (n)	N/A
	Requests/day (n)	N/A
Offline mode		Yes
Software		No
Local parameterization possible		No
	Developer	No
	User	No
Users		
Primary care		Yes
Dental medicine		Yes
Hospital medicine		Yes
Surgical prophylaxis		Yes
Patients		
Children		Yes
Pregnant women		Yes
Breastfeeding		Yes
Chronic kidney disease		Yes
Informations		
Type of infection	Extensive list of infections caused by bacteria, viruses, parasites and fungi including :	

	Urinary tract infections<br>Genital infections<br>Upper respiratory tract infections<br>Lower respiratory tract infections<br>Skin and soft tissues infections<br>Digestive tract infections<br>Prophylaxis<br>Central nervous system infections<br>Cardiovascular infections<br>Bone and joint infections<br>Febrile neutropenia<br>Eye infections<br>Central venous catheter-related infections<br>Dental infections<br>Bloodstream infections
Systematic criteria	Site of infection<br>Nature of infection
Optional criteria	Additionnal information about infection, pathogen or patient
Type of information provided	
Antibiotic selection	Yes
Priority	Yes
Dose	Yes
Route of administration	Yes
Duration	Yes
Allergy	Yes
Side effects	Yes
Adaptation GFR	Yes
Prescription printing	No
Context and reminders	Yes
Locally adapted	No
Scientific sources cited	Yes
References	No
Description	N/A

Table S10. Characteristics of *Prescriptor.*

**Prescriptor**			
Administration			
Name			RFCLIN-PRIMAIR
	Funding	Nature	Public
		Name(s)	ARS Franche-Comté
Scientific references			Local and national guidelines from SPILF, CMIT, HAS, SFAR and scientific literature
Date of creation			2014
Updating		Last	March 2017
		Frequency	< yearly
Contact			jleroy@chu-besancon.fr
Access			
	Cost		Free
	Smartphone app		No
		iOS	No
		Downloads (n)	N/A
		Reviews (n)	N/A
		Rating (/5)	N/A

		Android	No
		Downloads (n)	N/A
		Reviews (n)	N/A
		Rating (/5)	N/A
	Website		Yes
	Website URL	https://guides-bua.azurewebsites.net/	
		Users/day (n)	N/A
		Requests/day (n)	N/A
	Offline mode		Yes
	Software		Yes
Local parameterization possible			No
		Developer	No
		User	No
Users			
Primary care			Yes
Dental medicine			No
Hospital medicine			Yes
Surgical prophylaxis			No
Patients			
Children			No
Pregnant women			Yes (incomplete)
Breastfeeding			No
Chronic kidney disease			No
Informations			
	Type of infection	Urinary tract infections<br>Infections in obstetrics and gynecology<br>Intra-abdominal infections<br>Central venous catheter-related infections<br>Antibiotic prophylaxis in pregnancy	
	Systematic criteria	Site of infection<br>Nature of infection	
	Optional criteria	Additionnal information about infection, pathogen or patient	
Type of information provided			
	Antibiotic selection	Yes	
	Priority	Inconstant	
	Dose	Yes	
	Route of administration	Yes	
	Duration	Yes	
	Allergy	Yes	
	Side effects	No	
	Adaptation GFR	No	
	Prescription printing	No	
	Context and reminders	Yes	
	Locally adapted	Yes	
	Scientific sources cited	Yes	
	References	No	
	Description	N/A	

Table S11. Characteristics of *Antibiothérapie Pédiatrique.*

**Antibiothérapie Pédiatrique**		
Administration		
Name		CHU Sainte-Justine
Funding	Nature	Unknown

Name(s)		Unknown
Scientific references		Local (mainly), national and international guidelines
Date of creation		2019
Updating	Last	August 2021
	Frequency	Monthly
Contact		ME@messil.com
Access		
Cost		19,99 € per year
Smartphone app		Yes
	iOS	Yes
	Downloads (n)	N/A
	Reviews (n)	N/A
	Rating (/5)	N/A
	Android	Yes
	Downloads (n)	N/A
	Reviews (n)	N/A
	Rating (/5)	N/A
Website		No
	Users/day (n)	N/A
	Requests/day (n)	N/A
Offline mode		Yes
Software		No
Local parameterization possible		No
	Developer	No
	User	No
Users		
Primary care		Yes
Dental medicine		Yes
Hospital medicine		Yes
Surgical prophylaxis		No
Patients		
Children		Yes (exclusively)
Pregnant women		No
Breastfeeding		No
Chronic kidney disease		No
Informations		
Type of infection		Extensive list of pediatric infections caused by bacteria, viruses, parasites and fungi including:<br>Neonatal infections<br>Urinary tract infections<br>Upper respiratory tract infections<br>Lower respiratory tract infections<br>Skin and soft tissues infections<br>Digestive tract infections<br>Prophylaxis<br>Central nervous system infections<br>Cardiovascular infections<br>Bone and joint infections<br>Febrile neutropenia<br>Eye infections<br>Central venous catheter-related infections<br>Dental infections
Systematic criteria		Nature of infection
Optional criteria		N/A

Type of information provided	
Antibiotic selection	Yes
Priority	Yes
Dose	Yes
Route of administration	Yes
Duration	Yes
Allergy	Yes
Side effects	Yes
Adaptation GFR	No
Prescription printing	No
Context and reminders	Yes
Locally adapted	Yes
Scientific sources cited	No
References	No
Description	N/A

Table S12. Characteristics of *AntibioHelp®*.

**AntibioHelp®**			
Administration			
Name			RaMiPA project (Raisonner pour Mieux Prescrire les Antibiotiques)
Funding		Nature	French National Agency for the Safety of Medicines (ANSM)
		Name(s)	Public
Scientific references			French and International Clinical Practice Guidelines<br><br>Clinical expertise (Dr Frederic Mechaï infectious disease consultant at Avicenne, and Dr Mounira Lafarge microbiologist consultant)
Date of creation			2020
Updating		Last	2020
		Frequency	N/A
Contact			rosy.tsopra@nhs.net
Access			
	Cost		Free
	Smartphone app		N/A
		iOS	N/A
		Downloads (n)	N/A
		Reviews (n)	N/A
		Rating (/5)	N/A
		Android	N/A
		Downloads (n)	N/A
		Reviews (n)	N/A
		Rating (/5)	N/A
	Website		Yes
	Website URL		https://antibiohelp.doctoproject.fr/login
		Users/day (n)	N/A
		Requests/day (n)	N/A
	Offline mode		No
	Software		No
	Local parameterization possible		No
		Developer	Yes

User	No
Users	
Primary care	Yes
Dental medicine	No
Hospital medicine	No
Surgical prophylaxis	No
Patients	
Children	Yes
Pregnant women	Yes
Breastfeeding	No
Chronic kidney disease	No
Informations	
Type of infection	11 infectious diseases among :<br>Urinary tract infections<br>Upper respiratory tract infections<br>Lower respiratory tract infections<br>Genital infections
Systematic criteria	Site of infection<br>Nature of infection<br>Patient profile (Age, gender, pregnancy)<br>Antibiotic properties
Optional criteria	Antibiotic allergy<br>Additional information about infection
Type of information provided	
Antibiotic selection	Yes
Priority	Yes
Dose	No (Soon)
Route of administration	Yes
Duration	No (Soon)
Allergy	Yes
Side effects	Yes
Adaptation GFR	No (Soon)
Prescription printing	No
Context and reminders	Yes
Locally adapted	No
Scientific sources cited	Yes
References	Tsopra, R. et al. Helping GPs to extrapolate guideline recommendations to patients for whom there are no explicit recommendations, through the visualization of drug properties. The example of AntibioHelp® in bacterial diseases. Journal of the American Medical Informatics Association 26, 1010–1019 (2019).<br><br>Tsopra, R., Lamy, J.-B. & Sedki, K. Using preference learning for detecting inconsistencies in clinical practice guidelines: Methods and application to antibiotherapy. Artif Intell Med 89, 24–33 (2018).<br><br>Tsopra, R., Jais, J.-P., Venot, A. & Duclos, C. Comparison of two kinds of interface, based on guided navigation or usability principles, for improving the adoption of computerized decision support systems: application to the prescription of antibiotics. J Am Med Inform Assoc 21, e107-116 (2014).

	Tsopra R, Mechaï F, Sedki K, Lamy JB. Antibio-Help®, an innovative clinical decision support system for improving antibiotic prescriptions in primary care – Newletter APUA (Alliance for the Prudent Use of Antibiotics) – Summer 2019, Vol 37, No 2.
Description	CDSS description<br>Design process
